# Boosting Memory by tDCS to Frontal or Parietal Brain Regions? A Study of the Enactment Effect Shows No Effects for Immediate and Delayed Recognition

**DOI:** 10.3389/fpsyg.2018.00867

**Published:** 2018-06-04

**Authors:** Beat Meier, Philipp Sauter

**Affiliations:** Institute of Psychology, University of Bern, Bern, Switzerland

**Keywords:** DLPFC, PPC, transcranial stimulation, electrical stimulation, proactive interference

## Abstract

Boosting memory with transcranial direct current stimulation (tDCS) seems to be an elegant way to optimize learning. Here we tested whether tDCS to the left dorsolateral prefrontal cortex or to the left posterior parietal cortex would boost recognition memory in general and/or particularly for action phrases enacted at study. During study, 48 young adults either read or enacted simple action phrases. Memory for the action phrases was assessed after a retention interval of 45 min and again after 7-days to investigate the long-term consequences of brain stimulation. The results showed a robust enactment effect in both test sessions. Moreover, the decrease in performance was more pronounced for reading than for enacting the phrases at study. However, tDCS did not reveal any effect on subsequent recognition memory performance. We conclude that memory benefits of tDCS are not easily replicated. In contrast, enactment at study reliably boosts subsequent memory.

## Introduction

Learning can be enhanced by doing. In fact, even the simulation of performing an action can enhance subsequent memory. This insight has been labeled the enactment effect and it has been reliably demonstrated empirically in various studies and populations (e.g., Cohen, 1981; Bäckman et al., 1986; Zimmer and Engelkamp, 1999; Knopf et al., 2005; Masumoto et al., 2015). The enactment effect occurs for all kinds of episodic memory tests such as free recall, cued recall, and recognition memory tests (Zimmer and Cohen, 2001, for a review). Recent evidence has suggested that stimulating the brain with a weak current can also enhance learning. In particular, transcranial direct current stimulation to the left dorsolateral prefrontal cortex (DLPFC) seems to be an elegant way to boost memory performance (e.g., Javadi and Walsh, 2012; Javadi et al., 2012; cf. Manenti et al., 2012; Leshikar et al., 2017). In the present study, the goal was to combine the enactment effect and tDCS stimulation to boost subsequent memory performance and to gain additional insight about the brain areas involved in the advantage of enactment.

While there is considerable agreement that motor and sensorimotor networks are involved during enactment encoding, functional magnetic imaging during retrieval has also suggested the involvement of parietal brain areas for retrieval of action phrases enacted at encoding (Russ et al., 2003). To follow up on the brain areas involved in the enactment effect and in episodic memory formation more generally, we compared anodal tDCS stimulation of the left dorsolateral prefrontal cortex (as in Javadi and Walsh, 2012) and anodal tDCS stimulation of the left posterior parietal cortex (as suggested by the fMRI results of Russ et al., 2003).

Through the application of a current between two electrodes (i.e., an anode and a cathode) tDCS can modulate cortical excitability (Nitsche and Paulus, 2000, 2001). Typically, anodal tDCS is thought to induce subthreshold membrane depolarization (Nitsche et al., 2003b; Bikson et al., 2004; Ruffini et al., 2013), and it has been suggested that tDCS modulates mechanisms of cortical metaplasticity which in turn modify the synaptic bonds between neurons (Nitsche et al., 2003a, 2004; Fritsch et al., 2010; Stagg et al., 2011). As tDCS modulates cortical plasticity and cortical plasticity is generally involved in learning, the application of tDCS has the potential to modulate learning and memory (Rioult-Pedotti et al., 2000; Liebetanz et al., 2002). However, the exact mechanisms of modulation which are induced by tDCS are not well understood yet (but see Jacobson et al., 2012b; Pellicciari et al., 2013; Romero Lauro et al., 2014; Pisoni et al., 2017 for recent progress).

While there is extensive research on tDCS of the motor cortex for motor functions (cf. Horvath et al., 2015; Savic and Meier, 2016), fewer studies have investigated episodic memory (cf. Dedoncker et al., 2016). Most of these studies have tackled the left DLPFC to enhance episodic memory. Moreover, several studies have found modulating effects of parietal cortex tDCS in episodic memory (Jacobson et al., 2012a; Schaal et al., 2013; Jones et al., 2014; Pergolizzi and Chua, 2015; Pisoni et al., 2015).

Most relevant for the present study, several previous studies have found a modulatory effect of tDCS to parietal areas on subsequent recognition memory. For example, Jones et al. (2014) systematically tested the effects of left vs. right posterior parietal cortex (PPC) anodal stimulation across four experiments. Their results revealed that only left hemispheric stimulation at encoding enhanced subsequent memory performance. Thus, for the present study, we also targeted the left PPC.

The design of the present study was inspired by the study of Javadi and Walsh (2012). They used a procedure that involved two study phases, one before stimulation (pre-stimulation) and one after stimulation (post-stimulation; in fact to be precise, stimulation started 15 min before the second study phase and continued until the end of the study phase). This allowed a within-subject comparison of stimulation effects by testing recognition memory after a 45 min stimulation wash-out retention interval. In their first experiment in which tDCS stimulation was varied at encoding, Javadi and Walsh tested the effects of anodal and cathodal stimulation of the left DLPFC against two control conditions (sham and M1). For anodal stimulation they found better subsequent recognition memory performance for post-stimulation trials. In contrast, for cathodal stimulation they found worse subsequent recognition memory performance for post-stimulation trials. No memory differences between pre- and post-stimulation trials were found for the control conditions. Thus, anodal left DLPFC stimulation boosted subsequent recognition memory. Here, we used a similar set-up with anodal stimulation of the left DLPFC. In addition we administered anodal tDCS to the left posterior parietal cortex (PPC) and a sham stimulation control condition. We hypothesized that DLPFC stimulation at encoding may boost recognition memory generally and independent of encoding condition. In addition, based on the fMRI results by Russ et al. (2003), parietal stimulation may particularly boost recognition of action phrases that were enacted at encoding.

Moreover, we included a delayed test session in which recognition memory was assessed again after 1 week. This allowed investigating potential long-term effects of tDCS on episodic memory consolidation. Moreover, it allowed testing the longevity of the enactment effect which, to our knowledge has not yet been addressed in the literature.

## Methods

### Participants

Forty-eight adults took part in the study (27 women). Their average age was *M* = 24.50, *SD* = 4.376 (range from 18 to 38), their average education was *M* = 15.92 years, *SD* = 2.766, and they all spoke German fluently. The study was approved by the faculty ethics committee and all participants gave written informed consent before the experiment. Participants were randomly assigned to one of the three stimulation conditions (DLPFC, PPC, sham).

### Design

The design was a 3 × 2 × 2 × 2 mixed-factorial with the tDCS stimulation (DLPFC, PPC, sham) manipulated between subjects and encoding (read, enact), stimulation phase (pre-stimulation, post-stimulation) and retention interval (immediate, delayed) manipulated within subjects.

### tDCS

**Figure 1:**
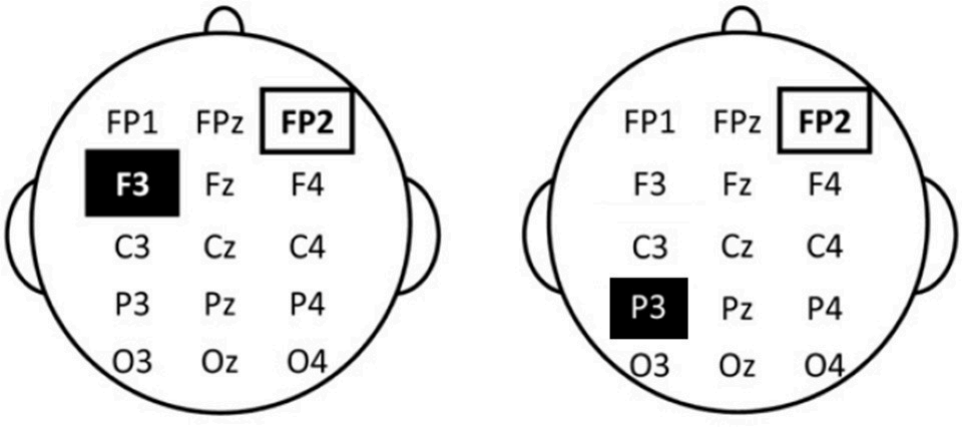
Schematic depiction of electrode positions. **Left**: Anodal stimulation of the left dorsolateral prefrontal cortex. **Right**: Anodal stimulation and Sham stimulation of the parietal cortex.

tDCS stimulation was based on the protocol of Javadi and Walsh (2012). Saline soaked sponge electrodes sized 30 × 30 mm for the target-electrode and 50 × 70 mm for the reference-electrode and a DC Brain Stimulator Plus device (NeuroConn, Ilmenau, Germany) were used. For prefrontal stimulation of the left DLPFC, the anode electrode was placed over F3 as in Javadi and Walsh. For parietal stimulation, the anode electrode was placed over CP3 as in Schaal et al. (2013) (cf. Mottaghy et al., 2002). For sham stimulation, we used the same CP3 electrode setup. For all three conditions, the cathode was placed over the right supraorbital area. Figure 1 shows a schematic presentation of the electrode positions in the different stimulation conditions. Stimulation was set at 0.8 mA to achieve approximately the same current density as Javadi and Walsh, who used a 35 × 35 mm sponge and 1 mA stimulation (resulting in a current density of 0.082 mA/cm^2^). Current density in the present study was 0.089 mA/cm^2^. Fade in and fade out was set to 5 s each. Duration of the stimulation varied across the three treatment-groups: Participants in the DLPF and PPC condition were stimulated for 1,200 s, while participants in the sham condition were stimulated for 30 s. As in Javadi and Walsh (2012), stimulation started after the first study phase and continued throughout the second study phase for a total of 20 min. Consistent with previous studies, the second study phase was labeled “post-stimulation” rather than “during stimulation” because the beginning of this study phase was post onset of stimulation. Participants were blind with respect to stimulation condition.

### Materials

#### Memory task

Stimulus materials consisted of 160 simple action phrases (e.g., flip a coin), adapted from Molander and Arar (1998). They were divided into 16 lists, which were counter-balanced across different phases of the experiment. For each participant, eight lists were presented during study (4 pre-, and 4 post-stimulation). For the immediate test, two pre-and two post-stimulation lists and four new lists were used. Similarly, for the delayed test, the other two pre- and post-stimulation lists and four other new lists were used. Thus, for each participant, half of the action pairs were presented once at study, and every action pair was presented once at test. Within each stimulation condition, the lists were counterbalanced across participants such that each of the 16 lists occurred equally often in each experimental phase. Each of these conditions was assigned to one participant in each stimulation group. Lists and materials are included in the Supplementary Materials 2.

#### Filler tasks

A demographic questionnaire, a vocabulary test (WST*;* Schmidt and Metzler, 1992), and a sustained attention test (d2-R; Brickenkamp et al., 2010) were used as filler tasks. In addition, American sitcom movie clips were used to attain the 45 min retention interval between study 2 and test 1.

### Procedure

**Figure 2:**
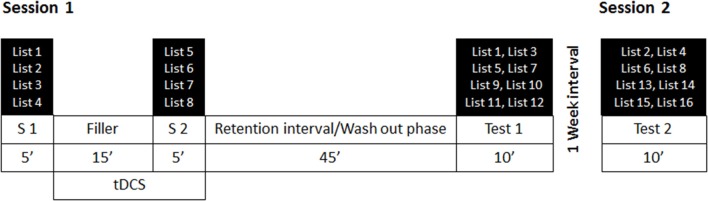
Procedure and exemplary depiction of the counterbalancing of the stimulus lists for each session. S1 = Study phase 1, S2 = Study phase 2. Four lists were used in each study phase (in this example, lists 1–5 and lists 5–8) and half of each were presented with read and enact instructions, respectively. Also two lists of each instruction condition, one from S1 and one from S2 were presented in an immediate or a delayed test phase, together with another four lists that were not presented previously (in this example, lists 9–12 and lists 13–16). Lists were counterbalanced across study conditions, instruction conditions, and test phases. Stimulation was initiated after the first study phase (S1) and continued until the end of the second study phase. Sham stimulation was stopped after 30 s of stimulation.

Participants performed the experiment individually under supervision of the experimenter. The experiment consisted of two sessions separated by 1 week. A schematic depiction of the procedure is presented in Figure 2.

Session 1 consisted of two study phases, one before and one after stimulation, a retention interval of 45 min to allow potential stimulation effects to wash out before testing, and a first, immediate, recognition test. Session 2 was scheduled a week later and consisted of the second, delayed, recognition test.

In session 1, participants were first informed about tDCS and the experimental procedure. After providing informed consent, the saline soaked sponges and electrodes were installed. Then, pre-stimulation encoding began, lasting for ~5 min without tDCS-treatment. Participants were informed that they will presented with short action phrases on the computer screen. Depending on the prompt on the screen they were instructed either to read the sentence (when the prompt “read” was presented above the action phrase) or to enact the action (when the prompt “enact” was presented above the action phrase). Each phrase was presented for six s. After the first study phase, tDCS-treatment was started and lasted for 20 min. During the first phase of tDCS stimulation, participants performed a set of filler tasks. After 15 min of stimulation, the post-stimulation study phase began, during which stimulation continued, following the same procedure as pre-stimulation. After a 45-min retention interval during which participants watched American sitcoms, the immediate recognition test was conducted. Participants were informed that they would be presented with another set of action phrases, some of which had been presented earlier in the experiment and that they have to decide for each phrase whether they had seen the sentence before. They were instructed to press the key b, if they recognized the sentence and the key n if they did not. After a positive decision they gave a remember/know response by pressing either the 1- or the 2-key. After a total of 80 trials (two “old” pre-stimulation lists, two “old” post-stimulation lists, and four “new lists, each consisting of 10 action phrases), session 1 ended with a reminder for the session 2.

Session 2 took place 1 week after the first session. No tDCS stimulation was administered. Only of the delayed recognition test was performed which followed the same procedure as in session 1, but with different the materials. That is, the 80 trials consisted of the other two “old” pre-stimulation lists, the other two “old” post-stimulation lists, and other four “new lists. After the recognition test, there was a detailed debriefing about the aim of the study.

### Data analysis

Analyses of variance (ANOVAs) were used for data analyses. As an index of recognition memory, we calculated the discrimination score Pr (hits minus false alarms; cf. Snodgrass and Corwin, 1988), which takes into account both hits and false alarms at the same time. Originally, we run additional analyses on Remember and Know-judgements. However, as these analyses did not reveal any additional insights or differences between tDCS stimulation and sham conditions, we do not report them in the Results section. However, these data are presented in the Supplementary Table 1. Analyses for Hits and False Alarms are also presented in the Supplementary Tables 2, 3. Effect sizes (η^2^) represent partial eta squared.

## Results

**Figure 3:**
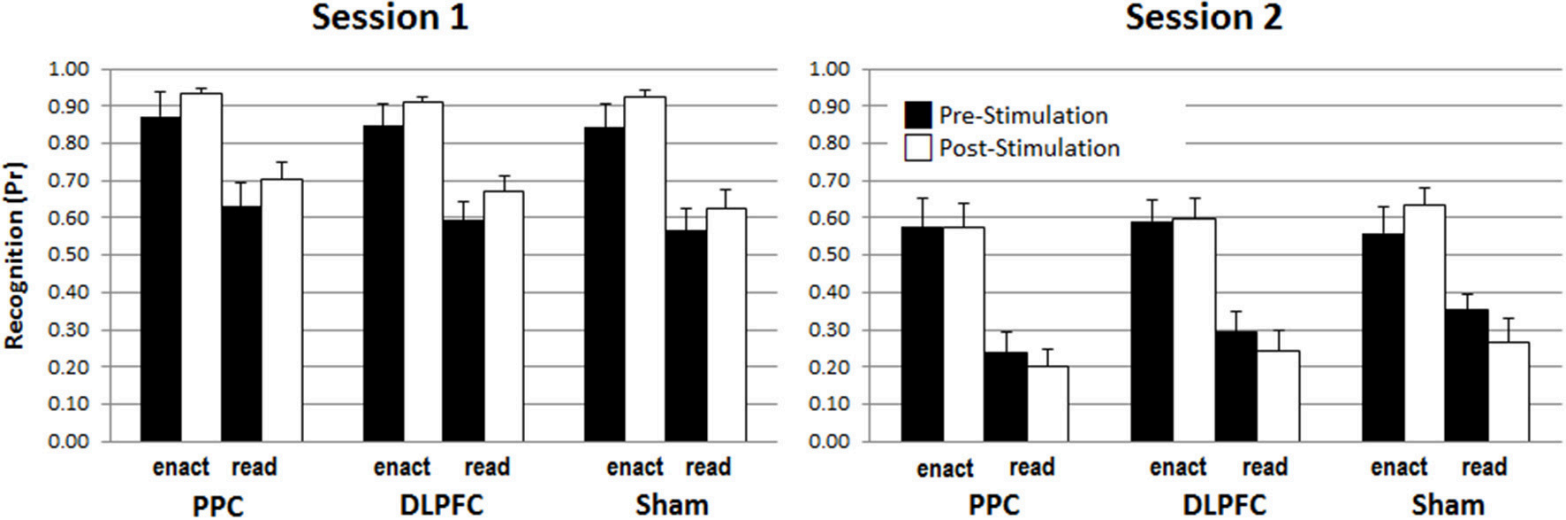
The enactment effect of recognition memory (*Pr*-scores) across stimulation conditions, pre-/post-stimulation phases and test sessions. Error bars represent standard errors.

To analyze the impact of tDCS stimulation (DLPFC, PPC, sham), encoding (read, enact), stimulation phase (pre, post) and retention interval (immediate, delayed) on recognition memory performance, a four-way mixed ANOVA was calculated on discrimination score *Pr*. These results are depicted in Figure 3.

The ANOVA revealed a main effect for retention interval, *F*_(1, 45)_ = 260.597, *p* < 0.001, η^2^ = 0.853, due to better performance in the immediate test, *M* = 0.76, *SD* = 0.11, compared to the delayed test, *M* = 0.42, *SD* = 0.14. There was also a main effect of enactment, *F*_(1, 45)_ = 139.550, *p* < 0.001, η^2^ = 0.756, due to better performance for enacted items, *M* = 0.74, *SD* = 0.14, compared to read items, *M* = 0.45, *SD* = 0.13. However, there was no effect of stimulation *F*_(2, 45)_ = 0.009, *p* = 0.991, or stimulation phase, *F*_(1, 45)_ = 0.618, *p* = 0.436. Most critically, there were also no interactions involving stimulation and stimulation phase, indicating that the application of tDCS did not modulate memory performance at all neither for read nor for enacted action phrases nor for immediate or delayed test, all *F*s < 1.190, *p*s > 0.313 (see Supplementary Table 4 Results for the specific values for the non-significant interaction effects). We addressed the power of these critical non-significant interactions involving tDCS-condition and stimulation phase using a post-hoc power analysis with G^*^Power (cf., Erdfelder et al., 1996). The largest effect size of the critical interactions involving stimulation and stimulation phase was η^2^ = 0.035 (i.e., the interaction between encoding, stimulation and stimulation phase) and the resulting statistical power was 0.38. To find a significant interaction with a power of 0.95, a sample size of at least 132 participants would have been necessary, as indicated by an additional a priori power analysis based on these empirical results.

**Figure 4:**
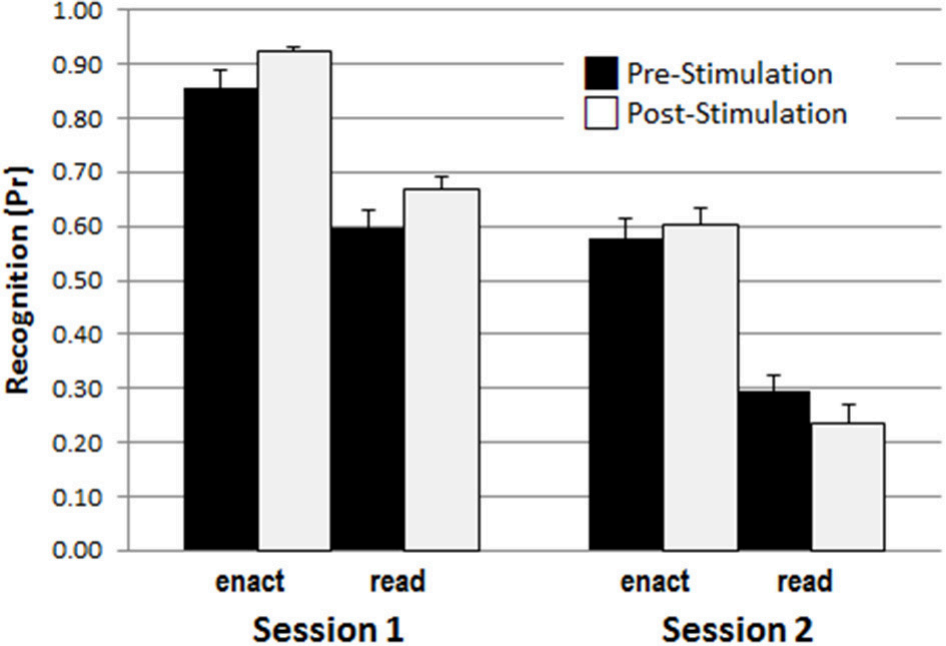
The enactment effect of recognition memory (*Pr*-scores) across test sessions, and pre-/post-stimulation phases, illustrating the interaction between retention interval, encoding condition, and stimulation phase.

In addition, the results showed a three-way interaction involving retention interval, encoding and study phase, *F*_(1, 45)_ = 4.211, *p* = 0.046, η^2^ = 0.086, and significant two-way interactions between retention interval and encoding, *F*_(1, 45)_ = 4.305, *p* = 0.044, η^2^ = 0.087, and between retention interval and stimulation phase *F*_(1, 45)_ = 9.398, *p* = 0.004, η^2^ = 0.159. To disentangle the triple interaction, two separate ANOVAs with the within-subject factors retention interval and encoding for both study phases, pre-stimulation and post- stimulation were conducted. For pre-stimulation, there were significant main effects for retention interval, *F*_(1, 48)_ = 120.631, *p* < 0.001, η^2^ = 0.715, and encoding, *F*_(1, 48)_ = 84.463, *p* > 0.001, η^2^ = 0.638, but no interaction between retention interval and encoding, *F*_(1, 48)_ = 0.336, *p* = 0.565, η^2^ = 0.007. In contrast, for post-stimulation, besides of the two main effects for retention interval, *F*_(1, 48)_ = 214.143, *p* < 0.001, η^2^ = 0.817, and encoding, *F*_(1, 48)_ = 123.960, *p* < 0.001, η^2^ = 0.721, the ANOVA revealed also a significant interaction of retention interval and encoding [*F*_(1, 48)_ = 8.662, *p* = 0.005, η^2^ = 0.153], indicating a larger decline for read items learned after the stimulation compared to enact items learned after the stimulation. This effect is illustrated in Figure 4.

As this effect was not affected by stimulation type it rather reflects an effect of proactive interference in the delayed condition. Specifically, while in the immediate test the shorter delay between the post stimulation study and the test phase was somewhat beneficial, possibly reflecting a kind of recency effect, this advantage disappeared and was even reversed after the 1 week retention interval. It is likely that this reversal occurred during consolidation reflecting stronger replay of action phrases from the first stimulation phase. Importantly, this effect was only observed for the read phrases which, compared to enacted phrases, were processed more shallowly.

However, as the main goal was to investigate the effect of tDCS on recognition memory performance and this effect did not materialize in the overall analysis we run some further analysis to exclude potential explanations. First, one might argue that the statistical power of the present study was not sufficient to find an interaction including the between-subject factor group. Importantly, however, the design of the study also allows a within-subject comparison of pre- vs. post stimulation. In the Javadi and Walsh study, which was based on *N* = 16, this comparison was significant with a strong effect size. A power analysis confirmed that with an alpha of 0.05, this design has sufficient power (1-Beta = 0.92) to detect an effect of *d* = 0.8 (Erdfelder et al., 1996). In the present study which also included *N* = 16 in each stimulation group, this effect was not significant, as indicated by an additional analysis that included only Session 1 in order to compare the effects of tDCS for each stimulation group separately. Specifically, the 2 × 2 ANOVAs with enactment (read vs. enact) and stimulation phase (pre-vs. post-stimulation) showed a significant main effect of enactment [with *F*s_(1, 15)_ of 28.351, 55.756, and 31.118, all *p*s < 0.001 for the PPC-, DLPFC- and Sham-stimulation groups, respectively), but no effect for stimulation phase [*F*_(1, 15)_ = 1.241, *p* = 0.283 for PPC, *F*_(1, 15)_ = 1.122, *p* = 0.306 for DLPFC, and *F*_(1, 15)_ = 1.097, *p* = 0.312 for sham]. Similarly, no significant enactment x stimulation phase emerged [*F*_(1, 15)_ = 0.067, *p* = 0.799 for PPC, *F*_(1, 15)_ = 0.155, *p* = 0.699 for DLPFC, and *F*_(1, 15)_ = 0.254, *p* = 0.621 for sham]. Thus, these further within-subjects analyses give no hint for any differential effects of the stimulation groups vs. the control condition. However, they corroborate the conclusion that, in any case, enactment is a stronger modulator of recognition performance than tDCS stimulation, at least with the stimulation protocols administered in the present study.

However, as performance in the enact condition was high, in particular in session 1, potential stimulation effects may have been overshadowed. In order to exclude this possibility, we run a further analysis for the read condition in Session 1 only. Again, we focused on the within-subject effect of stimulation phase (i.e., pre- vs. post-stimulation). Simple *t*-test for dependent variables resulted in *t*_(15)_ = 1.056, *p* = 0.308 for PPC, *t*_(15)_ = 1.047, *p* = 0.312 for DLPFC, and *t*_(15)_ = 0.747, *p* = 0.466 for sham. Thus, again, these analyses show no hint for a differential effect of stimulation on recognition performance. Rather they indicate that tDCS did not have a modulating effect on memory performance.

Last but not least, we tested this hypothesis further using Bayesian analysis. Bayes factors (*BF*) were used to assess the strength of evidence for H1 relative to H0 (Wagenmakers et al., 2015). A *BF* of above 3 indicates evidence for the alternative hypothesis and below 1/3 evidence for the null hypothesis. *BFs* between 3 and 1/3 indicate data insensitivity in distinguishing H0 and H1 (Dienes, 2014). Using JASP (Version 0.8.6.0; cf. Wagenmakers et al., 2015), we calculated a Bayesian mixed-factorial ANOVA with tDCS stimulation (DLPFC, PPC, sham) manipulated between subjects and encoding (read, enact), stimulation phase (pre-stimulation, post-stimulation) and retention interval (immediate, delayed) manipulated within subjects. The results are presented in Table 1. Most relevant are the Bayes Factors in the last column. The main effects of retention interval and enactment gave large Bayes Factors, indicating substantial evidence against the null hypothesis which is complementary to the highly significant effects in the traditional ANOVA presented above. In contrast, all the analyses relevant to the effects of tDCS, that is, effects involving the interaction between tDCS-condition and stimulation phase (pre vs. post) gave values close to zero, thus indicating substantial evidence in favor of the null hypothesis. Thus, the results of the Bayesian analysis support the conclusion that tDCS did not have an effect in this study.

**Table 1:** JASP output table of the Bayesian ANOVA on *Pr*-Rates with tDCS stimulation (DLPFC, PPC, sham) varied between-subjects and encoding (read, enact), stimulation phase (pre, post) and retention interval (immediate, delayed), all varied within-subjects.

**Effects**	**P(incl)**	**P(incl|data)**	**BF _Inclusion_**
tDCS	0.886	0.095	0.013
Interval	0.886	1.000	>100
Instruction	0.886	1.000	>100
PrePost	0.886	0.434	0.099
tDCS ^*^ Interval	0.503	0.027	0.027
tDCS ^*^ Instruction	0.503	0.004	0.004
tDCS ^*^ PrePost	0.503	0.002	0.002
Interval ^*^ Instruction	0.503	0.348	0.527
Interval ^*^ PrePost	0.503	0.243	0.317
Instruction ^*^ PrePost	0.503	0.094	0.102
tDCS ^*^ Interval ^*^ Instruction	0.120	0.000	<0.001
tDCS ^*^ Interval ^*^ PrePost	0.120	0.000	<0.001
tDCS ^*^ Instruction ^*^ PrePost	0.120	0.000	<0.001
Interval ^*^ Instruction ^*^ PrePost	0.120	0.006	0.043
tDCS ^*^ Interval ^*^ Instruction ^*^ PrePost	0.006	0.000	<0.001

*P(incl) = prior inclusion probability, P(incl|data) = posterior inclusion probability, BF _Inclusion_ = Bayes Factor (i.e., change from prior to posterior inclusion odds)*.

## Discussion

The aim of this study was to investigate whether tDCS would modulate the enactment effect in recognition memory. Besides enactment, we also varied the retention interval to investigate potential long-term effects. The results revealed a robust enactment effect in both test intervals. However, the decline across a 1 week interval was larger for action phrases that were only read compared to those that were enacted, and this decline was stronger for those phrases that were read in the second stimulation phase, administered after stimulation, than for those read in the first stimulation phase, administered before stimulation. However, as all these effects were not modulated by the application of tDCS, neither over DLPFC nor over PPC compared to sham stimulation, we conclude that tDCS stimulation was not the source of this effect, and overall, tDCS stimulation of the DLPFC and of the PPC with the current study protocol was not suitable to modulate memory performance.

This failure is somewhat surprising, as for DLPFC stimulation, we used a very similar stimulation protocol as Javadi and Walsh (2012) who were able to boost recognition memory performance. It is possible that the slight differences in electrode size and current intensity may be responsible for the lack of tDCS effects. They used a 35 × 35 mm anode sponge and 1 mA stimulation resulting in a current density of 0.082 mA/cm^2^. We used a 30 × 30 mm anode sponge with 0.8 mA stimulation resulting in a current density of 0.089 mA/cm^2^. Modeling studies of the motor cortex have shown that such differences would not yield much different outcomes (e.g., Miranda et al., 2009, Figure 4), but due to differences in tissue properties these result may not generalize to other cortical areas such as DLPFC or PPC. Thus, further modeling studies are required to exclude this explanation.

Another possibility is that differences in the materials and procedure were responsible for the different results. For example, at encoding the participants in the study of Javadi and Walsh had to respond to the number of syllables and imagine the words while we asked participants to read or enact action phrases. As sentences are more complex and involve both syntactic and morphologic processes, they also involve additional neural processing correlates. In fact, in episodic memory research many variables have been identified that affect memory performance consistently across many situations (i.e., materials, test formats etc.) such as manipulations of levels of processing, generation, or enactment, thus, we do not believe that these differences can be the cause for the different outcome of tDCS-stimulation. Specifically, as enacting read material engages presumably additional premotor and posterior parietal areas, we would still have to find differential effects between the PPC and sham stimulation condition. It is also possible that the verbal filler task may have had a specific effect on the tDCS conditions. So far, no study has addressed the influence of a spoken video filler task on tDCS.

Another possibility is that recognition memory is less susceptible to DLPFC stimulation than cued recall. This interpretation is compatible with the results of a recent study by Leshikar et al. (2017) who found a performance benefit for cued recall but not recognition even after a 24 h retention interval. On the other hand, a recent study by de Lara et al. (2017) also failed to boost memory performance in a verbal-associative learning task using a multi-electrode DLPFC tDCS montage. Importantly, similar to Leshikar et al. (2017), they tested cued recall either immediately or after 24 h and did not find any beneficial effects on memory performance after stimulation.

Similar explanations can be discussed in relation to the null-effects of PPC stimulation. For example, Schaal et al. (2013) who found a beneficial effect of PPC tDCS on pitch memory used somewhat higher current strength (i.e., 2 mA). Jones et al. (2014) who found a beneficial effect of PPC tDCS on the California Verbal Learning test (CLVT) stimulated their participants at a somewhat more posterior brain area (P3 electrode). Also, the CVLT involves several study trials and tDCS may be more effective when applied repeatedly to the same study material. Jacobson et al. (2012a) also stimulated at the P3 electrode position during a word learning task similar to Javadi and Walsh (2012). They also found a beneficial effect when they compared anodal left PPC stimulation to cathodal right PPC stimulation. Thus, again one might argue that methodological differences between the studies are responsible for the different results of the present study.

However, more probable, the inconsistency to find modulating effects of tDCS may root in the variability of the cortical changes caused by tDCS. This variability may be at the core of the still insufficient reliability of tDCS as a method to boost memory performance. In addition to better understand the effects of different stimulations, more systematic explorations of potential effects of methodological differences both on the level of experimental tasks and on the level of stimulation parameters are necessary to advance the field (cf. Savic et al., 2017a,b).

As long as there is no better understanding of the conditions under which tDCS works reliably, a fruitful research strategy may be to include an experimental manipulation into the study design that can provide interesting results beyond the potential effects of tDCS stimulation. In the present study, we followed such a strategy by testing the trajectory of the enactment effect across 1 week. In fact, this is the first study that has addressed this question and besides of the failure to find any beneficial effect of tDCS on memory performance, the results of this study provide strong evidence that enacting at study gives memory a long-lasting boost (cf. Meier et al., 2013, for a similar effect of word-frequency).

To conclude, on a more general level, and related to the goal to enhance memory performance, one may argue that—as demonstrated in the present study—simple findings from experimental psychology such as the instruction to enact at encoding are more reliable tools to boost memory performance than tDCS stimulation of the brain.

## Ethics statement

This study was approved by the Ethics committee of the human science faculty of the University of Bern.

## Author contributions

BM designed the study. PS collected the data and analyzed the data. BM and PS wrote the paper.

### Conflict of interest statement

The authors declare that the research was conducted in the absence of any commercial or financial relationships that could be construed as a potential conflict of interest.
