# Supplementary material for: Boosting Memory by tDCS to Frontal or Parietal Brain Regions? A Study of the Enactment Effect Shows No Effects for Immediate and Delayed Recognition
Source: Front Psychol. 2018 Jun 4;9:867. doi: 10.3389/fpsyg.2018.00867 (PMC5994422; doi:10.3389/fpsyg.2018.00867)

Supplementary Material

Boosting memory by tDCS to frontal or parietal brain regions? A study of the enactment effect shows no effects for immediate and delayed recognition

Beat Meier* & Philipp Sauter

*** Correspondence:** Beat Meier: beat.meier@psy.unibe.ch

# Table 1

Detailed description of recognition memory parameters hits and false alarms, separately for remember and know judgements, across retention interval (immediate, delayed), encoding condition (read, enact), study phase (pre-stimulation, post-stimulation), and stimulation condition (left dorsolateral prefrontal cortex, left posterior parietal cortex, sham)


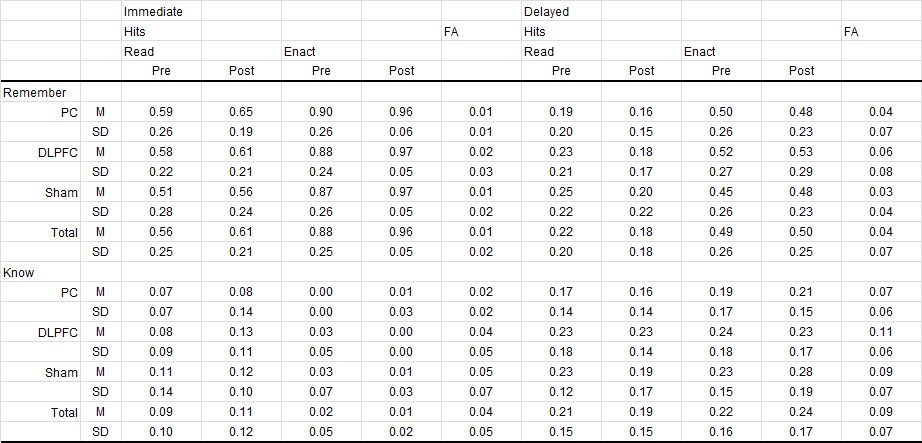


# Table 2

Hit rates were analyzed with a four-way mixed Analysis of Variance (ANOVA) with tDCS stimulation (DLPFC, PPC, sham) varied between-subjects and encoding (read, enact), stimulation phase (pre, post) and retention interval (immediate, delayed), all varied within-subjects.


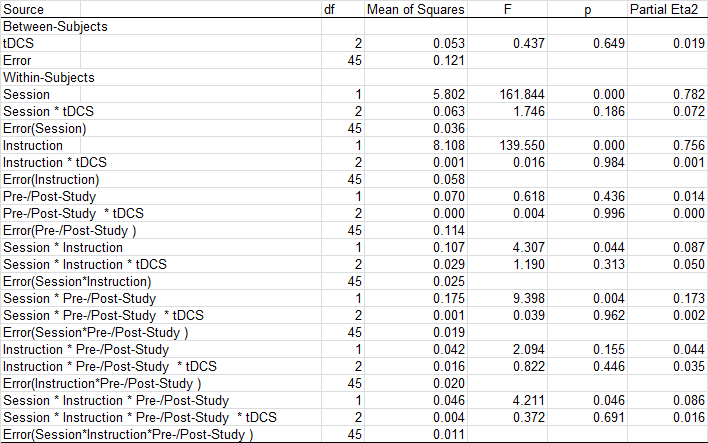


# Table 3

False Alarm rates were analyzed with a two-way mixed Analysis of Variance (ANOVA) with tDCS stimulation (DLPFC, PPC, sham) varied between-subjects and retention interval (immediate, delayed) varied within-subjects. Note that False Alarms do not carry any information that is related to the experimental conditions as it is not possible to have new pre- vs. post stimulation trials or new read vs. enact trials.


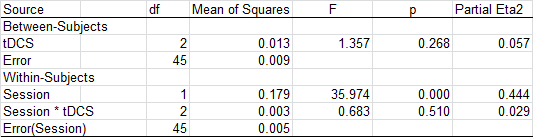


# Table 4

Pr-rates were analyzed with a four-way mixed Analysis of Variance (ANOVA) with tDCS stimulation (DLPFC, PPC, sham) varied between-subjects and encoding (read, enact), stimulation phase (pre, post) and retention interval (immediate, delayed), all varied within-subjects.


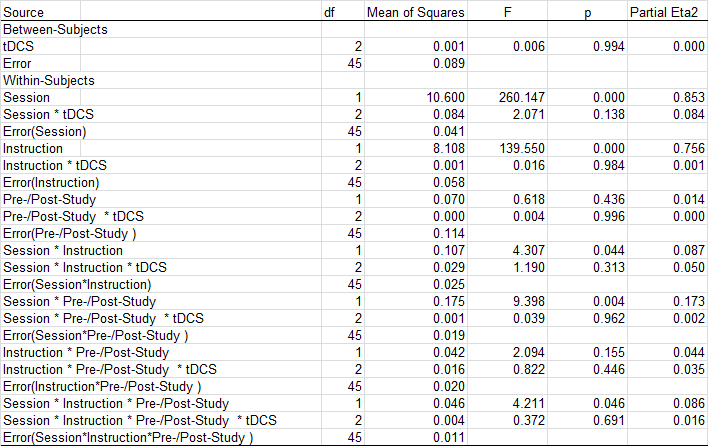

Supplement: Supplementary file 1 [file Table_1.DOCX]
