# Supplementary material for: Boosting Memory by tDCS to Frontal or Parietal Brain Regions? A Study of the Enactment Effect Shows No Effects for Immediate and Delayed Recognition
Source: Front Psychol. 2018 Jun 4;9:867. doi: 10.3389/fpsyg.2018.00867 (PMC5994422; doi:10.3389/fpsyg.2018.00867)
Supplement: Supplementary file 2 [file Table_2.DOCX]

**Supplementary Materials**

Item Sentence

L1Item1 In Buch blättern

L1Item2 Blumen pflücken

L1Item3 Holz spalten

L1Item4 Papier zerreissen

L1Item5 Sauce rühren

L1Item6 Staubsaugen

L1Item7 An Tür klopfen

L1Item8 Schlafend stellen

L1Item9 Schirm öffnen

L1Item10 Fliege einfangen

L2Item1 Finger kreuzen

L2Item2 Stirn runzeln

L2Item3 Ohren zuhalten

L2Item4 Tür aufschliessen

L2Item5 Arme verschränken

L2Item6 Glocke läuten

L2Item7 Dreimal blinzeln

L2Item8 Wasserkrug auffüllen

L2Item9 Sonnenlotion einschmieren

L2Item10 Auf Tisch schlagen

L3Item1 Hände falten

L3Item2 Mit Fingern schnippen

L3Item3 Katze füttern

L3Item4 Laut gähnen

L3Item5 Korkenzieher benutzen

L3Item6 Mit Fuss stampfen

L3Item7 Hund streicheln

L3Item8 Münze werfen

L3Item9 Kreis in Luft malen

L3Item10 Schneeball formen

L4Item1 Feuerzeug anmachen

L4Item2 Ins Mikrophon singen

L4Item3 Tube auspressen

L4Item4 Augen verdrehen

L4Item5 Pendel schwingen

L4Item6 Knopf zuknöpfen

L4Item7 Spaghetti aufrollen

L4Item8 Kuss schicken

L4Item9 Zigarette rauchen

L4Item10 Mantel aufhängen

L5Item1 Autofahren

L5Item2 Butter auf Brot schmieren

L5Item3 Loch in Münze bohren

L5Item4 Zunge rausstrecken

L5Item5 Aus einem Glas trinken

L5Item6 Mit Nastuch winken

L5Item7 Schläfen reiben

L5Item8 Pille schlucken

L5Item9 Bild zeichnen

L5Item10 Telefon abnehmen

L6Item1 Fenster öffnen

L6Item2 Konfetti schneiden

L6Item3 Kurbel drehen

L6Item4 Baseballschläger halten

L6Item5 Pistole abfeuern

L6Item6 Haare schamponieren

L6Item7 Kerne aus Kürbis entfernen

L6Item8 Deckel auf Pfanne legen

L6Item9 Kissen stopfen

L6Item10 Murmel rollen

L7Item1 Puzzle zusammenstellen

L7Item2 Schulterklopfer geben

L7Item3 Durch Mikroskop schauen

L7Item4 Bleistift spitzen

L7Item5 Zeit von der Armbanduhr ablesen

L7Item6 Salzstreuer benutzen

L7Item7 Kaffee rühren

L7Item8 Kopfhaare glatt streichen

L7Item9 Briefumschlag zukleben

L7Item10 Bonbon auspacken

L8Item1 Sich verbeugen

L8Item2 Brett an Tisch lehnen

L8Item3 Suppe essen

L8Item4 Mund abwischen

L8Item5 Wurst schneiden

L8Item6 Salutieren

L8Item7 Vor Kälte zittern

L8Item8 Mit Bausteinen spielen

L8Item9 Beine ausstrecken

L8Item10 Fingerring anziehen

L9Item1 Stange hinaufklettern

L9Item2 Brief frankieren

L9Item3 Teig kneten

L9Item4 Nase putzen

L9Item5 Auf Pferd reiten

L9Item6 Ball werfen

L9Item7 Brett an Wand nageln

L9Item8 Teller trocknen

L9Item9 In Tastatur schreiben

L9Item10 Tombola ziehen

L10Item1 Telefonnummer wählen

L10Item2 Etikett abziehen

L10Item3 Billet vorweisen

L10Item4 Teller jonglieren

L10Item5 Zigarette ausmachen

L10Item6 Zitrone ausdrücken

L10Item7 Roulette spielen

L10Item8 Feder in Tinte tunken

L10Item9 Ohrfeige geben

L10Item10 Bluse bügeln

L11Item1 Leicht Lächeln

L11Item2 Pullover anziehen

L11Item3 Chor dirigieren

L11Item4 Glühbirne auswechseln

L11Item5 Durch Feldstecher gucken

L11Item6 Finger zählen

L11Item7 Lippen ablecken

L11Item8 Knopf drücken

L11Item9 Stecker aus ziehen

L11Item10 Brille aufsetzen

L12Item1 Pfeilbogen schiessen

L12Item2 Deo auftragen

L12Item3 Messer schleifen

L12Item4 Spiegelbild betrachten

L12Item5 Fenster putzen

L12Item6 Auf Lippe beissen

L12Item7 Orange schälen

L12Item8 Schiebetüre schliessen

L12Item9 Uhr aufziehen

L12Item10 Zustimmend nicken

L13Item1 Nase kratzen

L13Item2 Stift balancieren

L13Item3 Zündholz zerbrechen

L13Item4 Kind in Armen wiegen

L13Item5 Wange kneifen

L13Item6 Armdrücken

L13Item7 Nasenspitze antippen

L13Item8 Wort ausradieren

L13Item9 Schnürsenkel binden

L13Item10 Lupe benutzen

L14Item1 Drachen steigenlassen

L14Item2 TV-Sender wechseln

L14Item3 Nägel feilen

L14Item4 Mit Rute Angeln

L14Item5 Zahnseide benutzen

L14Item6 Banane schälen

L14Item7 Reisverschluss öffnen

L14Item8 Handschuhe anziehen

L14Item9 Fenster einschlagen

L14Item10 Jemanden kratzen

L15Item1 Basketball dribbeln

L15Item2 Zeitung lesen

L15Item3 Schultern zucken

L15Item4 Im Stuhl zurücklehnen

L15Item5 Bei Prüfung abschreiben

L15Item6 Gitarre spielen

L15Item7 Teebeutel eintunken

L15Item8 Drink mixen

L15Item9 Geschirr waschen

L15Item10 Aufrecht sitzen

L16Item1 An Blume riechen

L16Item2 Durch Lippen pfeifen

L16Item3 Kopf schütteln

L16Item4 In Apfel beissen

L16Item5 Beine übereinanderlegen

L16Item6 Karte ziehen

L16Item7 Mit Finger drohen

L16Item8 In Tasse pusten

L16Item9 Dosenöffner benutzen

L16Item10 Autostopp machen

*Note.* L for List, 16 Lists with 10 Items each were created for counterbalancing
